# Fabrication of Multiple-Channel Electrochemical Microneedle Electrode Array via Separated Functionalization and Assembly Method

**DOI:** 10.3390/bios14050243

**Published:** 2024-05-13

**Authors:** Xin-Shuo Huang, Shuang Huang, Shan-Tao Zheng, Bao-Ming Liang, Tao Zhang, Wan Yue, Fan-Mao Liu, Peng Shi, Xi Xie, Hui-Jiuan Chen

**Affiliations:** 1 State Key Laboratory of Optoelectronic Materials and Technologies, School of Electronics and Information Technology, Sun Yat-sen University, Guangzhou 510006, Chinahuangsh239@mail.sysu.edu.cn (S.H.); zhengsht7@mail2.sysu.edu.cn (S.-T.Z.); liangbm@mail2.sysu.edu.cn (B.-M.L.); 2 School of Biomedical Engineering, Shenzhen Campus of Sun Yat-sen University, Shenzhen 518107, China; zhangt293@mail2.sysu.edu.cn; 3 School of Materials Science and Engineering, Sun Yat-sen University, Guangzhou 510006, China; yuew5@mail.sysu.edu.cn; 4 Division of Hypertension and Vascular Diseases, NHC Key Laboratory of Assisted Circulation and Vascular Diseases (Sun Yat-sen University), The First Affiliated Hospital, Sun Yat-sen University, Guangzhou 510080, China; liufm9@mail.sysu.edu.cn; 5 Department of Biomedical Engineering, City University of Hong Kong, Hong Kong SAR, China; pengshi@cityu.edu.hk

**Keywords:** micro/nanoneedle array, biosensing, blood glucose monitoring, separated functionalization and assembly, multi-channel electrochemical microneedle electrode array

## Abstract

Real-time monitoring of physiological indicators inside the body is pivotal for contemporary diagnostics and treatments. Implantable electrodes can not only track specific biomarkers but also facilitate therapeutic interventions. By modifying biometric components, implantable electrodes enable in situ metabolite detection in living tissues, notably beneficial in invasive glucose monitoring, which effectively alleviates the self-blood-glucose-managing burden for patients. However, the development of implantable electrochemical electrodes, especially multi-channel sensing devices, still faces challenges: (1) The complexity of direct preparation hinders functionalized or multi-parameter sensing on a small scale. (2) The fine structure of individual electrodes results in low spatial resolution for sensor functionalization. (3) There is limited conductivity due to simple device structures and weakly conductive electrode materials (such as silicon or polymers). To address these challenges, we developed multiple-channel electrochemical microneedle electrode arrays (MCEMEAs) via a separated functionalization and assembly process. Two-dimensional microneedle (2dMN)-based and one-dimensional microneedle (1dMN)-based electrodes were prepared by laser patterning, which were then modified as sensing electrodes by electrochemical deposition and glucose oxidase decoration to achieve separated functionalization and reduce mutual interference. The electrodes were then assembled into 2dMN- and 1dMN-based multi-channel electrochemical arrays (MCEAs), respectively, to avoid damaging functionalized coatings. In vitro and in vivo results demonstrated that the as-prepared MCEAs exhibit excellent transdermal capability, detection sensitivity, selectivity, and reproducibility, which was capable of real-time, in situ glucose concentration monitoring.

## 1. Introduction

In modern clinical medicine, the continuous detection of correlated physiological indicators within patients constitutes the development of new technologies for disease diagnoses and treatment [1,2]. The fluctuation of physiological indicators is closely associated with the progression of diseases, and real-time tracking of substance levels within the patient’s body aids in predicting disease development, facilitating an early diagnosis, and preventive measures [3,4,5]. Simultaneously, the continuous detection of the target fluctuation within patients plays an important role in assessing treatment efficacy and adjusting treatment plans [6,7,8]. By implanting sensing electrodes into the patient’s body, it is possible to trace the fluctuations of specific biomarkers, thereby achieving tracking of correlated physiological indicators [9,10,11]. With technological advancements, implantable electrodes can not only monitor and record electrical signals within tissues but also be utilized for therapeutics through electrical signal stimulation [12]. For example, pacemakers constructed by implanting electrodes in a patient’s heart can monitor heart beats, detect abnormal heart rates, and deliver electrical stimulation to maintain normal heart function when abnormalities occur [13]. Furthermore, short-term stimulation or long-term recording of brain activity can be achieved by affixing flexible implantable electrodes to the surface of a patient’s cerebral cortex. This may be employed for treating conditions such as epilepsy, Parkinson’s disease, and Alzheimer’s disease, among others in certain cases [8,14]. In addition to physical signal recording such as electrophysiology, implantable electrochemical electrodes modified with enzymes, antibodies, and other recognition elements can detect metabolites or proteins in situ [15]. Implantable electrochemical electrodes offer valuable tools for the continuous monitoring of biochemical signal fluctuations [16]. Presently, an invasive continuous glucose monitoring (CGM) device is one of the most mature applications of implantable electrodes [9]. Multi-layer modified implantable glucose electrodes, when embedded subcutaneously, can measure glucose concentrations in subcutaneous tissue fluid, thereby estimating blood glucose levels and effectively alleviating patients’ self-blood-glucose-management burden [17]. Implantable CGM electrodes enable continuous tracking of glucose fluctuations in interstitial fluid, gaining widespread recognition clinically and commercially [18,19,20].

However, the development of implantable electrochemical electrodes also faces several challenges [21,22,23]. Firstly, due to a relatively long length, implantable electrochemical electrodes are prone to nerve contact, leading to pain and bleeding with the risk of infection. Second, an allogeneic reaction that leads to tissue fibrosis and inflammation may be induced due to poor biocompatibility during long-term implantation, which can lead to signal distortion, sensitivity reduction, or a decrease in delivery directly. In recent years, microneedle (MN) arrays within the range of 500–800 μm have been invented to penetrate the stratum corneum of the skin and contact interstitial fluids [24,25,26]. By avoiding contact with subcutaneous nerves, microneedles can reduce the risk of capillary damage and the possibility of bleeding or pain, leading to a wide range of applications in biosensing and drug delivery [27,28,29].

Particularly, microneedle-based glucose monitoring has gained significant traction in diabetes detection and treatment [30,31,32]. For example, Gu et al. employs hyaluronic acid to encapsulate insulin and glucose oxidase to prepare glucose-responsive microneedle patches. At elevated glucose levels, the microneedle patches are transformed automatically, undergo dissociation, and release insulin in response to high glucose levels [33]. Similarly, Li et al. utilize porous materials to fabricate highly permeable microneedle arrays to penetrate the skin and form physical channels. Iontophoresis was combined to extract glucose from interstitial fluid for enzyme-catalyzed electrochemical detection, which enables in vivo blood glucose measurement [34]. In addition, modifying sensing electrodes with microneedles is also an effective approach in in vivo biomarker detection. Gao et al. integrated conductive polymers and nanomaterials to develop a microneedle electrode sensing array, which pierces the skin and facilitates the in-situ detection of glucose, uric acid, and cholesterol in interstitial fluids [35]. Furthermore, Yang et al. developed a microneedle array combined with iontophoresis-assisted insulin drug delivery [36]. By puncturing the skin, microscopic holes are formed and iontophoresis is used to promote insulin diffusion from the back of the microneedle into the interstitial fluids, enabling blood glucose regulation in vivo.

The development of multi-channel sensing technologies might further contribute to the development of microneedle-based electrochemical sensing arrays [37,38,39,40]. First, multi-channel sensing devices possess excellent spatial resolution to understand the biomarker distribution in the target area, thus contributing to early diagnoses and understanding the biochemical distribution in tissue. Second, the data collected from each channel can be utilized to gain the average signals, which can be combined with software algorithms to enhance detection stability and data reliability, reducing errors due to environmental interference at a specific skin location. Additionally, the development of multi-parameter detection devices based on multi-channel sensors allows for the targeted recognition of different biomolecules through different sensing channels, enabling multimodal recording of relevant indicators. This is beneficial to better understand the biochemical indicator profiles in relation to the disease [41,42,43].

However, the development of microneedle-array-based multi-channel electrochemical sensing arrays is currently constrained [27,44,45,46]. Electrochemical sensing arrays in biosensing primarily relies on the utilization of enzymes, achieving biomarker detection in interstitial fluid through microneedle electrode functionalization. However, the preparation method encounters challenges in producing multi-channel arrays on a small scale or developing versatile electrodes. The fine structure and small spacing of individual microneedle tips result in a lower spatial resolution for electrochemical sensing array functionalization. The partitioning modification of different materials on the same microneedle patch poses technical challenges in terms of processing complexity. The direct preparation of multi-channel electrochemical sensing arrays via three-dimensional micro-nanofabrication involves a complex manufacturing process. Additionally, commonly used silicon-based or polymer-based three-dimensional microneedle patches suffer from issues such as poor conductivity, simple structure and simple materials compositions, and insufficient mechanical hardness, making it difficult to meet the requirements of preparing multifunctional microneedle sensing electrodes for system integration [47,48,49].

**Figure 1:**
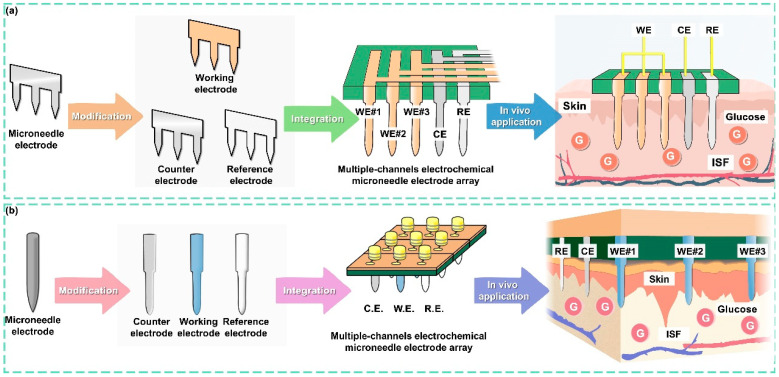
Illustration showing the strategy for MCEMEA fabrication based on microneedle components. (**a**) Development of the 2dMN-based MCEMEA. The 2d microneedle electrodes were fabricated using laser micromachining and modified individually to obtain 2dMN-based working electrodes, counter electrodes, and reference electrodes. Subsequently, through a separated functionalization and assembly process, the 2dMN-based MCEA for interstitial fluid glucose detection was developed. (**b**) The fabrication of the 1dMN-based MCEA. The 1dMN-based electrodes were prepared using laser micromachining and pre-positioned into a 1dMN-based electrode array. Multi-channel sensing of glucose concentration in subcutaneous tissue was achieved by the separated functionalization of the working electrode, counter electrode, and reference electrode, respectively, which were then positioned and assembled into the 1dMN-based MCEA.

In this study, we developed a separated functionalization and assembly process to fabricate multiple-channel electrochemical microneedle electrode arrays (MCEMEAs) based on 1D and 2D microneedle electrodes. The real-time, in situ detection of glucose levels in living animals was achieved while overcoming the difficulties of preparing and modifying microneedle sensing arrays (Figure 1). Using micro-nanofabrication, stainless steel sheets were developed into 2d microneedle (2dMN)-based electrode sheets, which were then functionalized individually to construct 2dMN-based sensing electrodes. Through parallel arrangement with the support structure, the 2dMN-based electrodes were assembled into a 2dMN-based multi-channel electrochemical array (MCEA), avoiding damage to the functionalized coatings by the high temperature during fabrication. For the 1d microneedle (1dMN)-based MCEA, 1dMN-based electrodes were prepared using laser cutting and protective layer deposition before combining with support structures to construct 1dMN-based electrode arrays. After the separated functionalization of the 1dMN-based electrodes, the electrodes were positioned for 1dMN-based MCEA assembly, which could reduce mutual interference and improve the stability during modification. In vitro simulation experiments verified the high skin penetration, and excellent detection stability and selectivity of the as-prepared MCEMEAs. In vivo experiments confirmed that the as-prepared MCEMEAs can detect the glucose concentration of living animals in real-time. This study is expected to promote continuous, stable, and reliable monitoring of relevant indicators in patients with diabetes and provide strong support for the development of multi-parameter detection systems for related diseases.

## 2. Results and Discussion

To address the challenges of microneedle-based multi-channel electrochemical sensing arrays in terms of materials, preparation, and functionalization, 1dMN-based and 2dMN-based MCEMEAs were constructed and their ability in skin penetration and in vivo glucose detection was verified. A 2dMN-based MCEA was constructed using 2D laser processing and individual sheet enzyme modification methods. The 2dMN-based electrode sheets were prepared by laser machining on stainless steel sheets, and then electroplating of gold was applied on the surface of each 2dMN-based electrode sheet to minimize the substances’ interference during detection. Generally, stainless steel contains components such as carbon, chromium, cobalt, nickel, etc., which are prone to electrode polarization during electrochemical sensing and cause signal interference. By modifying the surface of the stainless-steel electrode with gold, the direct contact between the stainless-steel electrode and the detection solution is isolated, which helps to improve the biocompatibility and anti-interference ability of the electrodes. In addition, the electrochemical deposition of platinum and dip-coating of glucose oxidase (GOx) were sequentially performed on the electrode surface to form the 2dMN-based working electrode, which could detect glucose concentration in the solution. Finally, after modifying the 2dMN-based counter electrode and the 2dMN-based reference electrode, the electrodes were assembled in a parallel arrangement to form a multi-channel electrochemical microneedle electrode array with three sensing channels, avoiding mutual interference between adjacent electrodes during fabrication. Similarly, the construction of the 1dMN-based MCEA relied mainly on one-dimensional processing and modification methods. Gold electroplating was applied to the laser-cut 1dMN-based electrodes by electrochemical deposition, after which the electrodes were pre-positioned with a 3D-printed resin. Next, 1dMN-based working electrodes were prepared by platinum electrochemical deposition and glucose oxidase modification, while the 1dMN-based reference electrode and 1dMN-based counter electrode were constructed by silver/silver chloride ink decoration or platinum electrochemical deposition on the pre-positioned 1dMN-based electrodes, respectively. Finally, the 1dMN-based electrodes were re-positioned and assembled to form an MCEMEA with three sensing channels, avoiding the damage to the functionalized layer of 1dMN electrodes.

**Figure 2:**
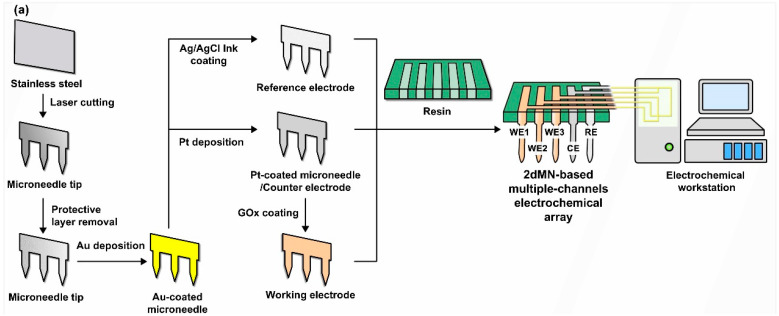

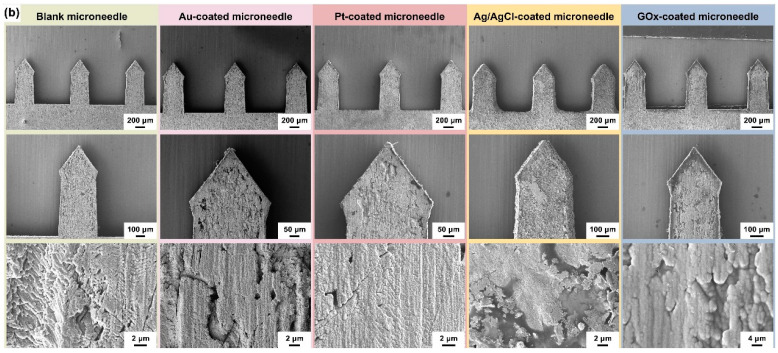
(**a**) The schematic diagram of the fabrication process of the 2dMN-based MCEA. Stainless steel sheets were laser cut for the fabrication of 2dMN-based electrode sheets. Then, the oxide layer of the electrodes was removed, and Pt deposition and GOx decoration were performed to form the 2dMN-based working electrode, counter electrode, and reference electrode. Finally, the electrodes were developed into the MCEMEA by the separated functionalization and assembly process in combination with a 3D-printed support base. (**b**) Surface morphology of 2dMN-based electrodes during fabrication at different scales. From left to right: the untreated 2dMN-based electrode; gold-electroplated 2dMN-based electrode; 2dMN-based counter electrode; 2dMN-based reference electrode; and 2dMN-based working electrode.

For the fabrication of the 2dMN-based MCEA, a laser cutting system was initially employed to cut stainless steel sheets, forming the 2dMN-based electrode sheet (Figure 2a and Figure S1). Subsequently, the 2dMN-based electrodes were cleaned prior to removal of the oxide layer, and then modified with multiple functional layers to obtain the 2dMN-based working electrode, counter electrode, and reference electrode, respectively. The 2dMN-based working electrode was constructed after Pt electrodeposition and GOx coating on the gold-electroplated 2dMN-based electrode (Figures S2 and S3). Glucose oxidase catalyzed the decomposition of glucose to produce H_2_O_2_, while the platinum layer further catalyzed the decomposition of H_2_O_2_, which caused an amperometric signal change. Platinum-coated 2dMN-based electrode sheets were used as counter electrodes, while 2dMN-based electrode sheets modified with silver/silver chloride were used as reference electrodes (Figure S4). After the fabrication of the 2dMN-based working electrode, counter electrode, and reference electrode, each electrode sheet was embedded into a 3D-printed resin base (Figure S5), and then adhered and encapsulated using PDMS (polydimethylsiloxane), resulting in an MCEMEA via a stacking process. This separated functionalization and assembly process could effectively mitigate potential mutual interference issues during the chemical modification.

The surface morphology of the 2dMN-based electrode during different modification steps was characterized, using a field emission scanning electron microscope (SEM). Due to the limited conductivity of the enzyme-modified microneedle electrodes, a thin layer of gold (approximately 10 nm thick) was deposited on the electrode surface via sputtering to enhance conductivity for electron microscope characterization. As shown in Figure 2b, each 2dMN-based electrode processed a length of 800 μm, a width of 400 μm, and a maximum width of about 440 μm. The microneedle tip with an angle of nearly 70° was sufficient to penetrate the stratum corneum and contact the interstitial fluid, avoiding contact with vessels or causing pain. There were about 20° chamfers on both sides of the needle tip, which helped to improve the microneedle adhesion after insertion, ensuring stable signal collection. The distance between the tips of each microneedle was about 1.2 mm, which was beneficial for stable microneedle array construction. The 2d blank microneedle was constructed based on stainless steel with excellent conductivity; only scratches and pits on the surface could be observed. After acid washing and electrochemical gold deposition, the pits on the electrode surface were covered and no visible bulge was found. After electrochemical platinum plating, the electrode conductivity was improved and the electrode surface was uniform and smooth, with fewer undulations. The thickness and surface morphology of the gold layer could be well adjusted by controlling the parameters of electrochemical deposition. The uniformity of the platinum-coated microneedle surface could be controlled by adjusting the current magnitude or the deposition duration during the fabrication. After electrochemical deposition, 2dMN-based electrodes showed no significant change in electrode diameter and morphology, which was still able to achieve stable skin puncture. The surface of the electrode modified by Ag/AgCl showed more lamellar structures and covered the electrode surface well, keeping the potential stable during in vitro and in vivo detection. After being modified by glucose oxidase (GOx), the platinum layer on the electrode surface was completely covered with some undulations. Due to the limited conductivity of the enzyme, the uneven coating of the gold layer showed obvious dark areas. The uniformly covered glucose oxidase helps to rapidly convert glucose in the detection and enable the electrochemical detection of H_2_O_2_.

**Figure 3:**
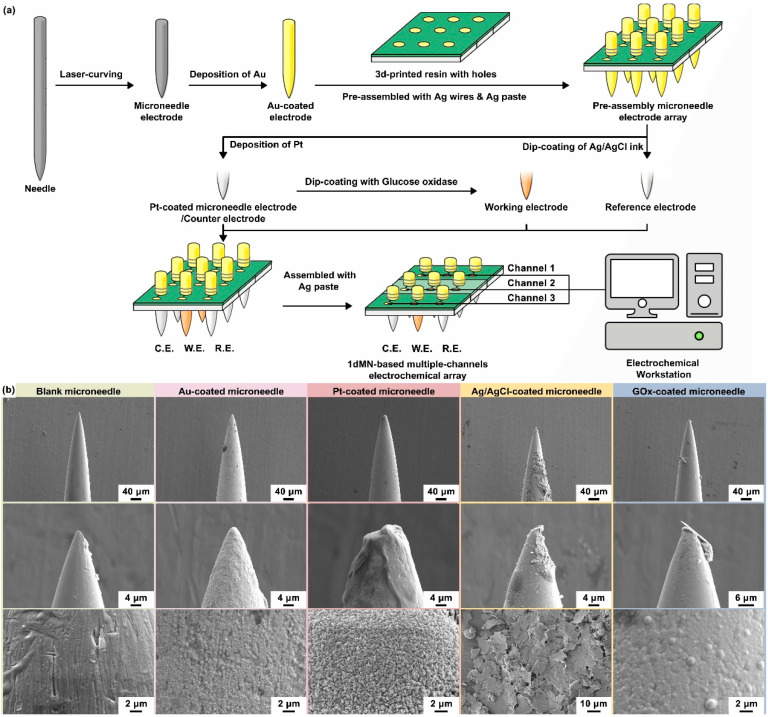
(**a**) The schematic of the 1dMN-based MECA fabrication process. The 1dMN-based electrodes were fabricated by laser cutting and pre-positioned by combining them with 3D-printed support structures and electrode pads. Subsequently, each microelectrode was modified separately to form the 1dMN-based working electrode, counter electrode, and reference electrode. Finally, the electrical signal channels were constructed through the processes of needle pulling, silver wire wrapping, silver adhesive bonding, and insulating coating. In this way, the 1dMN-based electrodes were integrated into the 1dMN-based MCEA via the separated functionalization and assembly process, which alleviates the mutual interference during modification. (**b**) Surface morphology of 1dMN-based electrodes during fabrication at different scales. From left to right: the untreated 1dMN-based electrode; gold-electroplated 1dMN-based electrode; 1dMN-based counter electrode; 1dMN-based reference electrode; and 1dMN-based working electrode.

Similarly, 1dMN-based electrodes were fabricated based on the separated functionalization and assembly process, for the construction of the 1dMN-based MCEA (Figure 3a). For 2dMN-based electrodes, each microneedle was interconnected via a metal substrate, making it impossible to construct isolated electrical signal channels on each microneedle tip. Multi-channel sensing arrays were prepared using 1dMN-based electrodes so that each microneedle became an addressable independent channel. First, the 1dMN-based electrodes were prepared by cutting acupuncture needles with a laser cutter and removing the oxide layer on the needle surface. The 1D needle was then electroplated with gold. Subsequently, a 3D-printed resin substrate was fabricated with through-holes according to the size of the microneedle diameter. At the same time, microelectrode arrays with three parallel electrical channels were designed and prepared on the surface of the polyimide (PI) substrate, where the pads were soldered with silver wires as extended electrical leads to connect the microneedle electrodes (Figures S6 and S7). 

The center of each microelectrode pad processed a through-hole, which allowed the microneedle electrode to pass through the pin-hole. A PDMS buffer layer with a thickness of about 3 mm was prepared, and attached underneath the resin substrate to adjust the length of the exposed microneedle tips. The 1dMN-based electrodes were sequentially inserted into the through-hole of the PI substrate, and the resin substrate, and penetrated through the PDMS buffer layer to form a 3 × 3 microneedle electrode array. At this period, the 1dMN-based electrodes were not fully immobilized and remained length-adjustable. The length of the needles exposed below the substrate was more than 3 mm, which was more convenient for the individual modification of the needle electrodes. Subsequently, the 1dMN-based counter electrode was fabricated through platinum electrochemical deposition on the gold-plated needle electrode, while the 1dMN-based reference electrode was obtained by coating with silver/silver chloride ink on the gold-electroplated needle electrode. The 1dMN-based working electrode was obtained by dip-coating with GOx on the surface of Pt-coated needle electrodes. During the detection process, GOx on the surface of the 1dMN-based working electrode would catalyze the decomposition of glucose in the solution, generating H_2_O_2_. Then, driven by an electrochemical bias, the generated H_2_O_2_ would be catalytically decomposed on the platinum surface, forming amperometric signals that correlate the glucose concentration with the collected electrical signal.

After the microneedle electrode modification, the length of the needle below the substrate was adjusted to be less than 1 mm, forming the 1dMN-based MCEA. To further improve the stability of the sensing circuit, silver wires were connected onto the back of the 1dMN-based electrodes using silver paste, and finally encapsulated by PMMA. As shown in Figure S8, the as-prepared 1dMN-based MCEA possessed a total of nine microneedles’ tip in a 3 × 3 array profile, and each microneedle was about 800 μm in length. The working electrodes contained three separated channels, resulting in three independent glucose detection channels. The reference electrode and counter electrode each possessed three connected microneedle electrodes as one electrical channel. These three working electrodes shared a counter electrode and a reference electrode, forming a multi-channel sensing array with independently addressable working channels. Subsequently, SEM was utilized to characterize the surface morphology of the 1dMN-based electrodes. As shown in Figure 3b, the length of each 1dMN-based electrode was approximately 800 μm and the diameter was approximately 200 μm, which was sufficient to achieve safe and stable skin penetration as well as interstitial fluid detection. The distance between the microneedle tips in the 1dMN-based MCEA was approximately 3 mm, which could be adjusted as needed to develop multi-channel or multifunctional sensing arrays. Based on stainless steel, 1d blank microneedles processed well electrical conductivity and mechanical strength. The surface morphology of the blank microneedle electrode was uniform, with only scratches and grooves visible in some areas. After acid washing and electrochemical gold deposition, the pits on the surface of 1d blank microneedles were evenly covered, and the conductivity was significantly improved. After electrochemical platinum deposition, more spiny structures could be observed on the electrode surface, due to the long deposition time and high electric field intensity. The formed spiny structure was beneficial to increase the specific surface area of the electrode and improve the detection sensitivity. The thickness and surface morphology of the gold and platinum layers could be adjusted by controlling the electrode area, current, or deposition time. The Ag/AgCl ink could evenly cover the electrode surface, and the laminated sheet material could be observed in the magnified image. After dip-coating of GOx, the platinum layer with a spiny structure was completely covered, and the electrode surface was relatively uniform and smooth with a small number of point-like protrusions. The uniform coverage of glucose oxidase could respond quickly to glucose fluctuation, and the 1dMN-based MCEA after decoration still processed well mechanical properties and could achieve stable skin penetration and biomarker detection.

Next, the electrochemical detection performance of the 2dMN-based MCEA was characterized in a glucose solution in vitro. The fasting blood glucose concentration in a healthy human body is generally in the range between 3.3 mmol/L and 6.9 mmol/L. Therefore, the glucose concentration in the in vitro test was gradually increased from 0 mmol/L to 12 mmol/L to cover the range of blood glucose levels in humans. A polyvinyl chloride film was used to simulate the skin stratum corneum, and the film covered the surface of a 25 mL beaker containing 20 mL of a PBS (phosphate buffer solution). The 2dMN-based working electrode was penetrated through the film so that the electrode tip was immersed in the solution. The performance of the 2dMN-based working electrodes was evaluated via CHI760E with the sensing electrode as the working electrode, the commercial platinum electrode as the counter electrode, and the commercial silver/silver chloride electrode as the reference electrode. In the electrochemical tests, i-t amperometric characterization was performed by applying a bias voltage (0.5 V vs. silver/silver chloride electrode) on the surface of the 2dMN-based working electrode. In the presence of oxygen, GOx on the electrode surface would catalyze the decomposition of glucose, producing gluconic anhydride and H_2_O_2_. Next, H_2_O_2_ was catalyzed to decompose and electron transfer occurred, generating an electrical signal that flowed into the electrodes, forming a current loop. In this way, the glucose in the solution was converted into H_2_O_2_, which was catalytically decomposed by the platinum layer of the microneedle electrode to generate a change in the current signal. Therefore, the presence of glucose concentration in the solution could be converted into a measurable amperometric signal. To minimize experimental errors, the recording was paused for 30 s each time for the glucose concentration to be increased during the experiments, to allow an adequate diffusion of the added glucose in the solution, after which the signal acquisition was resumed.

**Figure 4:**
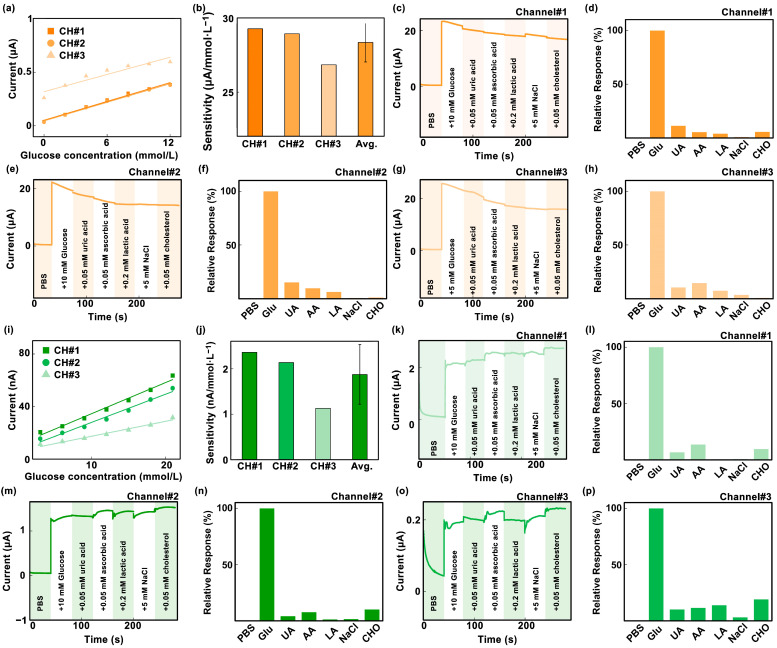
(**a**) Response curves of three sensing channels of the 2dMN-based MCEA to different glucose concentrations (0 to 12 mmol/L). (**b**) The sensitivity and data analysis of the three sensing channels of the 2dMN-based MCEA in glucose detection. (**c**–**h**) Current signals collected over time by the 2dMN-based MCEA in the presence of substances (glucose, uric acid, ascorbic acid, lactic acid, sodium chloride, and cholesterol) in the in vitro simulated solution, and the normalized results. The interferences of substances on the collection of amperometric signals were minimal. These figures correspond to the detection results of channel 1 (**c**,**d**), channel 2 (**e**,**f**), and channel 3 (**g**,**h**), respectively. (**i**) Response curves of three sensing channels of the 1dMN-based MCEA to different glucose concentrations (3 to 21 mmol/L). (**j**) The sensitivity and data analysis of the three sensing channels of the 1dMN-based MCEA in glucose detection. (**k**–**p**) Amperometric signals collected over time by the 1dMN-based MCEA in the presence of substances (glucose, uric acid, ascorbic acid, lactic acid, sodium chloride, and cholesterol) in the in vitro simulated solution, and normalized results. The substances’ interferences to data collection were minimal. These figures correspond to the detection results of channel 1 (**k**,**l**), channel 2 (**m**,**n**), and channel 3 (**o**,**p**), respectively.

As shown in Figure 4a, with the gradual increase in glucose concentration from 0 mmol/L to 12 mmol/L, the current detected by the 2dMN-based working electrode gradually increased from 0.038 μA to 0.38 μA in channel #1, with a detection sensitivity of 29.3 nA/mM, whereas, in channel #2, the current detected gradually increased from 0.033 μA to 0.38 μA, with a detection sensitivity of 28.9 nA/mM; in channel #3, the current detected increased from 0.26 μA to 0.60 μA, with a detection sensitivity of 26.8 nA/mM, and the average value of detection sensitivity for these three channels was 28.3 nA/mM (Figure 4b). In vivo glucose detection is mainly performed by electrochemical methods, which is easily interfered with by internal or external substances such as uric acid, L-ascorbic acid, lactic acid, salt, and cholesterol. The coexistence of multiple biomarkers may interfere with glucose detection, so the detection specificity of the 2dMN-based working electrode was evaluated based on the ratio of various interfering substances to glucose in healthy human blood. The specificity of the 2dMN-based working electrode was tested by sequentially adding 10 mmol/L of glucose, 0.2 mmol/L of uric acid, 0.05 mmol/L of ascorbic acid, 0.1 mmol/L of lactate, 5 mmol/L of the salt solution (represented by sodium chloride), and 2 mmol/L of cholesterol to the test solution during continuous monitoring. The results showed that for channel #1, a significant current signal change was detected in the presence of glucose (~22.0 μA), whereas the presence of other interfering substances, including uric acid, ascorbic acid, lactic acid, sodium chloride, or cholesterol, produced weak current signals (<2.5 μA) to the electrode, which were more than 8 times lower than the signals produced by glucose (Figure 4c,d). To facilitate the visual analysis of the amperometric signal changes, signals collected were normalized with the signal change generated by the glucose as a reference value. The results showed that the addition of uric acid, ascorbic acid, lactic acid, sodium chloride, and cholesterol produces relative interferences of approximately 11%, 5.2%, 3.7%, 0.6%, and 5.4% to the glucose-induced current signal, respectively. Among them, ascorbic acid and cholesterol caused more interference, but neither exceeded 15%. 

Similarly, for channel #2, the addition of glucose induced a significant signal change (~20.3 μA), whereas the presence of other interfering substances induced tiny signal changes (≤3.0 μA), which were more than 6 times lower than the glucose signal (Figure 4e,f). Normalized results showed that the addition of uric acid, ascorbic acid, lactic acid, sodium chloride, and cholesterol produced relative interferences of about 15%, 9.6%, 6.1%, 0.5%, and 0.9% to the glucose-induced signal, respectively. Among these, ascorbic acid produced a greater interference of about 15%. Channel #3 produced a more pronounced change in the amperometric signal in the glucose presence (about 24.0 μA), and was insensitive to the presence of interfering substances (<3.5 μA), which were more than 6 times lower than the signal produced by glucose (Figure 4g). After normalization, the relative interferences to the collected amperometric signals were about 10%, 14%, 7.2%, 3.4%, and 0.6%, with ascorbic acid producing a larger interference of about 14% (Figure 4h). The results above indicated that all three channels of the developed 2dMN-based MCEA were capable of detecting glucose with good performance.

Similarly, the electrochemical detection performances of different channels in the 1dMN-based MCEA were evaluated. As depicted in Figure 4i, with the gradual increase in glucose concentration from 3 mmol/L to 21 mmol/L, the current detected by the 1dMN-based working electrodes increased from 1 nA to approximately 90 nA. Detection sensitivities for each channel were 2.36 nA/mM, 2.13 nA/mM, and 1.13 nA/mM, respectively, with an average of 1.87 nA/mM (Figure 4j). The sensitivity of channel #3 was comparatively lower than the other two channels, possibly attributed to some inconsistency during the manual fabrication or electrode decoration of electrodes. To improve the detection stability, the fabrication processing could be improved by replacing manual operations with automated production. For in vivo applications, it is necessary to normalize the signals to reduce the detection error between channels. Similarly, different concentrations of glucose, uric acid, ascorbic acid, lactate, saline (represented by sodium chloride), and cholesterol were added sequentially to the testing solution of 1dMN-based working electrodes. Channel #1 exhibited significant amperometric changes with glucose addition (approximately 1.2 μA), while other interfering substances generated weak amperometric signals (<0.12 μA), more than 10 times lower than the glucose signal (Figure 4k). Normalized results indicated that the relative interference introduced by uric acid, ascorbic acid, lactic acid, sodium chloride, and cholesterol was approximately 3.9%, 7.4%, 1.1%, 1.3%, and 9.9%, respectively (Figure 4l). Notably, interferences from ascorbic acid and cholesterol were slightly high, but none of them exceeded 10%. Likewise, in channel #2, glucose caused a noticeable amperometric change (approximately 0.14 μA), while other interfering substances induced changes that were more than 5 times lower (≤0.03 μA). The normalization analysis demonstrated that the relative interferences introduced by uric acid, ascorbic acid, lactic acid, sodium chloride, and cholesterol were 9.9%, 11.3%, 13.6%, 2.9%, and 18.9%, respectively (Figure 4m,n). Interference from ascorbic acid was relatively high at around 19%. In channel #3, glucose introduction resulted in a more pronounced change in the amperometric signal (approximately 2.0 μA), while sensitivity to the presence of interfering substances was less pronounced (<0.3 μA), more than 6 times lower. The relative interferences introduced by uric acid, ascorbic acid, lactic acid, sodium chloride, and cholesterol were about 6.6%, 13.5%, 0.4%, 0.5%, and 9.4%, respectively (Figure 4o,p). The reproducibility of the fabricated MCEA was carried out via the repeated i-t test in 5 days. After 5-day storages, the sensitivities of each 2dMN-based working electrode maintained 94%, 95%, and 81%, with an average of 90%. For the 1dMN-based working electrode, the sensitivities maintained 96%, 89%, and 80%, with an average of 88% (Figure S9). To enhance the life of MCEA, employing biocompatible materials like polyvinylpyrrolidone or polyvinyl alcohol to reduce mechanical damage during skin penetration could be helpful. It is also effective to store the sensor in a cryogenic container or in an environment filled with inert gas. By maintaining the glucose oxidase activity of the sensing electrode, the life of the sensor can be extended and long-term monitoring can be achieved. These results suggested that the three detection channels of the 1dMN-based MCEA can respond selectively to glucose fluctuations and were insensitive to interference from other substances.

**Figure 5:**
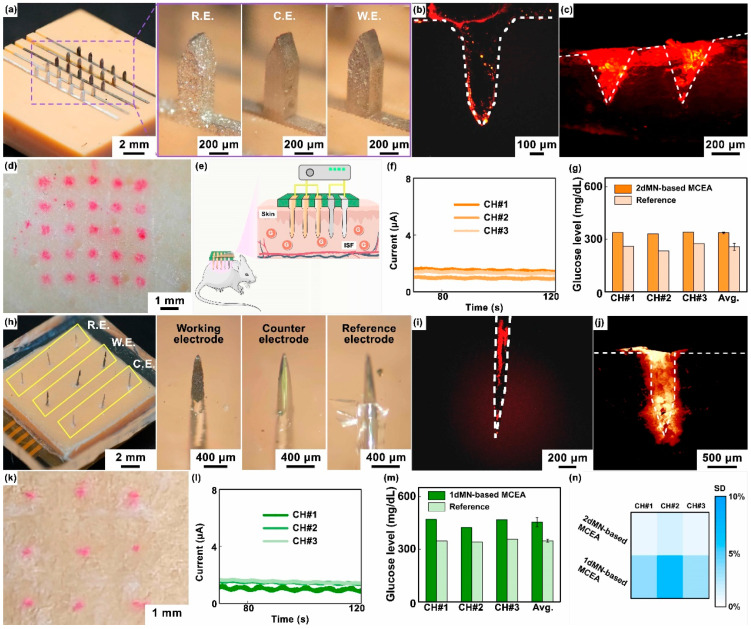
(**a**) Optical images of the 2dMN-based MCEA, including the 2dMN-based reference electrode, 2dMN-based counter electrode, and 2dMN-based working electrode. (**b**) The fluorescence image of the 2dMN-based electrode stained with Rhodamine B, observed under a fluorescence microscope. (**c**) The fluorescence characterization of Rhodamine B dye deposition within porcine skin after 2dMN-based electrodes’ penetration (cross-sectional view). (**d**) The optical image of the porcine skin surface staining after 2dMN-based MCEA penetration. (**e**) A schematic representation diagram showing the application of MCEMEA in vivo glucose detection in live rats. (**f**) In in vivo experiments, the 2dMN-based MCEA was inserted into rat skin and the amperometric signals collected in the three sensing channels were examined over time. (**g**) Results and the data analysis of glucose concentrations in interstitial fluid of living rats measured by three sensing channels in the 2dMN-based MCEA. All results were calculated from standard curves derived from in vitro detection and compared with reference values of blood glucose. (**h**) Optical images of the 1dMN-based MCEA, consisting of the 1dMN-based working electrode, 1dMN-based counter electrode, and 1dMN-based reference electrode, respectively. (**i**) The fluorescence image of the 1dMN-based electrode stained with Rhodamine B, observed via the fluorescence microscope. (**j**) The fluorescence characterization of Rhodamine B dye deposition within the porcine skin penetrated by 1dMN-based electrodes (cross-sectional view). (**k**) The optical image of the porcine skin surface staining after 1dMN-based MCEA penetration. (**l**) In vivo detection results of the three sensing channels in the 1dMN-based MCEA. The MCEA was inserted into the rat skin and the amperometric signals were examined over time. (**m**) Results and the analysis of the glucose concentrations in interstitial fluid of living rats measured by the 1dMN-based MCEA. The signals collected were converted according to standard curves and compared with blood glucose reference values. (**n**) The heatmap showing the standard deviation (SD) of different sensing channels of the MCEMEA in interstitial fluid glucose detection of living rats.

After 2dMN-based MCEA integration (Figure 5a), the transdermal performance of the 2dMN-based MCEA was investigated (Figure S10). The in vitro experiment was conducted using fresh porcine skin as a simulation of human skin to characterize the penetration ability of the 2dMN-based electrodes. First, a Rhodamine B solution was prepared, and the electrode tips were dipped into a small amount of the solution, uniformly staining the 2dMN-based electrodes. Subsequently, the 2dMN-based MCEA was placed on the surface of a fresh porcine skin, with pressure applied vertically from above to ensure the perpendicular insertion of the 2dMN-based MCEA into skin. After maintaining for 200 s, the 2dMN-based MCEA was withdrawn, and skin treated with microneedles was observed under an optical microscope and fluorescence microscope. 

Next, cross-sectional slices of porcine skin (approximately 200 μm thick) were obtained by cutting along the holes left by the 2dMN-based MCEA with a blade. Red fluorescence was observed under a fluorescence microscope. As shown in Figure 5b,c, tissues surrounding the transdermal site of the 2dMN-based MCEA emitted red fluorescence, demonstrating the successful skin penetration of the microneedle-based electrodes. Fluorescence microscopic images of the cross-section showed a clear deposition of the fluorescent dye, which was consistent with the morphology of the 2dMN-based electrodes. The maximum penetration depth of 2dMN-based electrodes was approximately 400 μm, shorter than the tip length of 2dMN-based electrodes (about 800 μm), most likely due to the skin elasticity that prevented complete embedding of the microneedles. The surface of the porcine skin penetrated by the 2dMN-based MCEA also exhibited the color correspondingly (Figure 5d). The total thickness of the stratum corneum (10 to 15 μm) and the epidermis (50 to 100 μm) of human skin is less than the penetration depth of the 2dMN-based electrode, and these results validated the skin-penetrating ability of the 2dMN-based MCEA. The excellent mechanical strength of the metal material enhanced the upper limit of the force exerted by the microneedles when penetrating the skin.

Subsequently, in vivo experiments were conducted on rats to verify the detection capability of the 2dMN-based MCEA (Figure 5e). First, the rats were depilated using surgical scissors and depilatory cream to expose the back’s skin. Then, 2dMN-based MCEAs were pressed onto the rats’ back skin, ensuring the microneedles’ penetration to the stratum corneum. A bias voltage of 0.5 V (vs. silver/silver chloride electrode) was applied to the 2dMN-based working electrodes, and the amperometric signal between the working and counter electrodes was recorded. To stabilize the signal, each sensing channel was acquired independently for 120 s by sharing the counter electrode and reference electrode. To reduce the dependence of the working electrode on the response time, the last 20 s of the stable amperometric signals was averaged and converted into glucose concentration correspondingly, based on the standard curve obtained in vitro. Simultaneously, blood samples were collected through the rats’ tail arteries to obtain reference values. Amperometric signals collected from the three sensing channels of the 2dMN-based MCEA were recorded separately and then converted to blood glucose levels. The results above confirmed stable electrical signals in all channels of the 2dMN-based MCEA during detection (Figure 5f). The detected subcutaneous glucose concentrations in rats were 339.0 mg/dL, 331.2 mg/dL, and 339.7 mg/dL, with an average of approximately 336.7 mg/dL (Figure 5g).

Similarly, transdermal performance was carried out after 1dMN-based MCEA fabrication (Figure 5h and Figure S11). As depicted in Figure 5i,j, the 1dMN-based electrode tip emitted red fluorescence under a fluorescence microscope after penetration, with an approximately 800 μm length. Cross-sectional images showed red fluorescence in the surrounding tissue, confirming the successful penetration of the 1dMN-based electrodes. In addition, the fluorescent dye deposition on the skin surface penetrated by the 1dMN-based MCEA was observed by microscopy, which closely matched the electrode distribution of the 1dMN-based MCEA (Figure 5k). Fluorescence microscopy results showed that the maximum penetration depth of the 1dMN-based electrodes was about 400 μm, and the average penetration depth was about 300 μm, which was more than the total thickness of the human epidermis (10 to 15 μm) and dermis (50 to 100 μm). These results confirmed the ability of the 1dMN-based electrodes to effectively penetrate the skin. The 1dMN-based electrodes were tested in vivo and the amperometric signals were recorded separately for each sensing channel for a duration of 120 s, suggesting that the 1dMN-based electrodes were able to maintain stable signal collection for more than 100 s in vivo (Figure 5l). The measured subcutaneous glucose concentrations in rats were 469.8 mg/dL, 423.1 mg/dL, and 467.9 mg/dL for the respective channels, with an average of 453.6 mg/dL (Figure 5m). To further assess the accuracy of different detection channels in MCEAs, the standard deviation was statistically analyzed using a heatmap (Figure 5n). For the 2dMN-based MCEA, the standard deviation for each sensing channel was 0.7%, 1.6%, and 0.9%, respectively. For the 1dMN-based MCEA, the standard deviation for each sensing channel was 3.5%, 6.7%, and 3.1%, respectively. For both the 1dMN-based MCEA and 2dMN-based MCEA, the standard deviation of all channels did not exceed 8%. Notably, for both the 1dMN-based MCEA and 2dMN-based MCEA, the amperometric signal tends to be stable after 20 s, and no intense transient could be found during measurement, suggesting that the dependencies of response on time of MCEAs are relatively low (Figure S12). By adjusting instrument parameters, modifying sensitive sensing materials, reducing the detection voltage, or optimizing software algorithms, the dependencies of response on time of the MCEAs could be further reduced.

In in vivo testing, no inflammatory response or bleeding was found in skin penetration and glucose sensing. To enhance the biocompatibility of the MCEA, biocompatible materials such as polyvinyl pyrrolidone can be used to decorate the sensing electrodes to prevent inflammatory reactions and reduce the risk of bleeding.

These results indicated that the as-prepared MCEAs could selectively measure glucose concentration in both in vitro and in vivo environments, and the detection sensitivity of each channel was relatively similar. Additionally, this separated functionalization and assembly process could be further extended to research applications for the detection of ions, reactive oxygen species, uric acid, and other indicators. Notably, the 1dMN-based MCEA was constructed based on isolated sensing electrodes, which could realize flexible electrode assembly as needed and was beneficial to the integration of multifunctional array devices. Based on the 1dMN-based MCEA, hollow microneedles or channel microneedles could also be integrated for subcutaneous drug delivery. However, the effective sensing area of the 1dMN-based system was limited and the amperometric signal may be affected. The detection sensitivity could be improved by combining signal amplification circuits or highly sensitive biometric components. The 2dMN-based MCEA was easy for preparation and the effective sensing area was increased. Meanwhile, the MCEA constructed based on planar microneedle electrodes processed higher mechanical strength and higher skin penetration capabilities for in vivo biomarker detection.

## 3. Materials and Methods

Preparation and modification of 2dMN-based electrodes: To fabricate the 2dMN-based electrode, the structural pattern of the 2dMN-based electrode was initially designed (Figure S1), followed by laser cutting on a stainless-steel sheet. The 200 μm thick SUS304 stainless steel sheet was cleaned with ethanol and dried at 80 °C. Subsequently, the YLP-F series fiber laser marking machine (Hanslaser Co., Ltd., Shenzhen, China) was employed for precise cutting of the stainless-steel sheet to obtain the 2dMN-based electrodes. The parameters were set as laser wavelength: 1.06 μm, engraving line speed: 1200 mm/s, power: 25 W, and cycles: 1500. The width and thickness of each microneedle sheet were 3 mm and 0.2 mm, respectively. The maximum width and length of each microneedle were 0.4 mm and 0.8 mm, respectively. Each 2dMN-based electrode had 5 distributed microneedles with a spacing of 1.2 mm. A handle, 15.4 mm in length and 1.5 mm in width, was connected to the back of the microneedle sheet for connection.

Next, the 2dMN-based electrode was treated with an acidic cleaning solution (containing 24% zinc oxide, 30% ammonium chloride, 6% hydrochloric acid, 30% acetic acid, 12% deionized water, and 3% surfactant; Yuncaitaotao Co., Ltd., Huizhou, China) to remove the oxide layer. Afterward, the electrode was washed in 80% ethanol and dried at 90 °C, then immersed in a gold sulfate solution (0.2 mmol/L; Yuncaitaotao Co., Ltd., Huizhou, China) for gold deposition (Figure S2). The introduction of gold is beneficial to enhance electrode stability under positive bias during electrochemical detection (Figure S3). By applying a continuous voltage of −0.8 V for 300 s, the uniform electrochemical deposition of gold was carried out on the microneedle electrode surface. The gold-electroplated microneedle electrode was further immersed in a platinum sulfate solution (1 mmol/L, Yuncaitaotao Co., Ltd., Huizhou, China) for platinum deposition. A voltage of about −0.3 V was applied for 600 s to electrochemically deposit platinum on the surface of the gold-plated microneedle electrode.

In preparing the glucose sensing electrode, a homogeneous mixture of 500 mL of bovine serum albumin (BSA, 80 mg/mL, Guangzhou Shuoheng Biotechnology Co., Ltd., Guangzhou, China), 200 mL of a glutaraldehyde solution (2.5% *w*/*w*, Sigma), and 100 mL of the glucose oxidase (GOx) solution (50 mg/mL, Sigma) was obtained. The mixture was uniformly coated on the surface of the platinum-plated 2dMN-based electrode and dried at room temperature overnight as a 2dMN-based working electrode. Meanwhile, the gold-electroplated 2dMN-based electrode was uniformly coated with silver/silver chloride ink (Yuncaitao Co., Ltd., Huizhou, China) and dried at 90 °C for 1 h to obtain the 2dMN-based reference electrode (Figure S4).

Assembly of 2dMN-based electrodes: For the integration of 2dMN-based electrodes, the supporting structure was designed using design software. A 15 × 15 mm resin (Figure S5) with a thickness of 3 mm was fabricated using a 3D printer (Jihe SE8K, DongGuan Broad Technology Co., Ltd., Dongguan, China). The 2dMN-based working electrode, counter electrode, and reference electrode were then arranged in parallel with a 3 mm spacing and embedded into the 3D-printed resin. Subsequently, PDMS was utilized to coat the back of the 2dMN-based electrode, simultaneously fixing the electrodes, and encapsulating the conductive part on the back of the electrodes to avoid short circuits. 

Fabrication and modification of 1dMN-based electrodes: Acupuncture needles (Jiuziling Pharmaceutical Co., Ltd., Anhui, China) were employed as the substrate for microneedle electrode fabrication. SUS304 stainless steel acupuncture needles were cut into 1dMN-based electrodes with a length of 6 mm using a YLP-F series fiber laser marking machine. The laser cutting conditions were as follows: laser wavelength—1.06 μm, engraving line speed—1000 mm/s, power—18 W, and engraving cycles—1200. Next, 1dMN-based electrodes were placed in an acidic washing solution (Yuncaitaotao Co., Ltd., Yunnan, China) to remove the oxide layer from the surface. Subsequently, after washing with ethanol and drying in an oven at 80 °C, a gold layer was electrodeposited onto the 1dMN-based electrodes. Using a gold electrode as the anode, the 1dMN-based electrode was connected to the cathode of the electrochemical workstation (CHI760E, CH Instruments Inc., Shanghai, China), and gold was electrodeposited in a gold sulfate solution. Then, the 1dMN-based electrodes were cleaned with ethanol and dried at 80 °C. For the fabrication of the substrate structure of the microneedle array, a hole-patterned resin was printed using a 3D printer, after which it was washed in ethanol and dried at 60 °C.

Pre-position of the 1dMN-based electrodes: The customized polyimide (PI) electrode (Figures S6 and S7, Shenzhen Honghong Precision Circuit Co., Ltd., Shenzhen, China) was laminated and patterned onto the hole-patterned support structure to achieve microneedle integration. The 0.5 mm thick silver wires were connected to the solder pad of the PI electrode, and a 3 mm buffer layer was prepared using polydimethylsiloxane (PDMS, Dow Corning DC 184). The 1dMN-based electrodes were sequentially inserted into the PI electrode, resin, and buffer layer, and their orientation and length were adjusted to ensure a stable position. Subsequently, silver wires soldered to the pads were wrapped around the 1dMN-based electrodes and further bonded with silver paste (Yuncaitaotao Co., Ltd.). The reserved length of the soldered silver wire allows the 1dMN-based electrode inserted into the substrate to remain unattached and fixed at this stage, thus providing an adjustable length for the 1dMN-based electrode under the substrate for further decoration.

Fabrication of 1dMN-based counter and reference electrodes: The 1dMN-based counter electrode was prepared by connecting the gold-electroplated 1dMN-based electrode to the cathode of the electrochemical workstation and plating it in the platinum sulfate solution for 1200 s with a commercial platinum electrode as the anode. To fabricate the 1dMN-based reference electrodes, the gold-electroplated 1dMN-based electrodes were uniformly coated with silver/silver chloride ink. The electrodes were then dried in an oven at 90 °C for 90 min and air-dried for 16 h at room temperature.

Fabrication of 1dMN-based working electrode: To fabricate the 1dMN-based working electrode, a mixture containing the glucose oxidase solution (50 mg/mL), BSA solution (60 mg/mL), and glutaraldehyde (2.5 mg/mL) at a volume ratio of 2:5:2 was prepared. The platinum-plated 1dMN-based electrode was coated with the mixture, uniformly pulled, and air-dried for 16 h at room temperature. Additionally, to maintain the activity of the sensing layer, the as-prepared electrode should be stored at 4 °C when not in use.

Assembly of 1dMN-based MCEA: The direction, position, and needle length of each electrode were adjusted to expose a uniform microneedle length of 800 μm beneath the support structure after the fabrication of the 1dMN-based electrodes. The electrodes were connected to the PI electrodes using silver-paste-coated and -bonded silver wires (Figure S8). A 2% PMMA (polymethylmethacrylate) solution was then applied to the surface of the PI electrode for insulation.

Surface morphology characterization of microneedle-based electrodes: The optical characterization of the prepared microneedle-based electrodes was carried out using a Minmax optical microscope. Meanwhile, the surface morphology of individual electrodes during the modification process was further characterized using SEM Pro (PHENOM, SCIENTIFIC). Due to the different electrical conductivity of each electrode, a thin layer of gold atoms (~10 nm thick) was deposited on the surface of the electrodes for observation.

Electrochemical performance characterization: For 2dMN-based electrodes, skin was simulated using a four-folded film and the film covered a 25 mL beaker to which 20 mL of the PBS (phosphate buffer solution) was added. The 2dMN-based electrodes were used to pierce the film to simulate the transdermal process. The 2dMN-based sensing electrodes were connected to the electrochemical workstation, with a commercial silver/silver chloride electrode as the reference electrode and a commercial platinum electrode as the counter electrode. The time–current curves of the sensing electrodes were collected over 300 s by immersing the electrodes in a PBS, applying a bias voltage (0.5 V vs. silver/silver chloride electrode) and gradually adding glucose to the PBS, with the glucose concentration varying from 0 mM to 12 mM. Similarly, for 1dMN-based electrodes, time–current curves of the 1dMN-based working electrodes were collected by applying a bias voltage (0.5 V vs. silver/silver chloride electrode), with the glucose concentration varying from 3 mM to 21 mM. After each experiment, the electrodes were stored in a 4 °C refrigerator to maintain the catalytic activity of oxidase. The reproducibility of the sensors was evaluated by the repeated i-t test every other day for 5 days.

Selectivity characterization of microneedle-based electrodes: Selectivity tests were performed by recording the time–current curves of the sensing electrodes through an electrochemical workstation with a series of selective tests in the PBS (phosphate buffer solution) including 10 mmol/L of glucose, 0.5 mmol/L of uric acid, 0.5 mmol/L of ascorbic acid, 0.2 mmol/L of lactic acid, 0.1 mmol/L of sodium chloride (representative of saline), and 0.5 mmol/L of cholesterol. The amplitude of the amperometric response was recorded for 300 s. In addition, to further analyze the signal changes caused by various disturbances, the value of amperometric change after glucose addition was set to 100% for normalization.

Penetration ability characterization of MCEMEA: A 2 mg/mL Rhodamine B solution was prepared and a small amount of the solution was absorbed onto a cotton swab, evenly coating the tip surface of the MCEMEA. Using fresh porcine skin as a substitute for human skin, the MCEMEA was placed vertically on the skin surface, and inserted tightly into the skin for a few minutes before removal (Figure S10). Subsequently, porcine skin was observed using an optical microscope. In addition, a razor blade was used to dissect along the cross-section of the microneedle electrode penetration site to obtain a thin slice (approximately 200 μm) after skin penetration. The skin slices were then observed under a fluorescence microscope for red fluorescence.

In vivo experiment of MCEMEA: This study was approved by the Sun Yat-sen University Animal Care and Use Committee. All animals received humane care according to institutional guidelines. SD-IGS rats were obtained from the Sun Yat-sen University Animal Laboratory for experimental research. The animals used included two diabetic rats. Initially, the rats were anesthetized using gas anesthesia, followed by the depilation of the rat’s back using surgical scissors and depilatory cream, resulting in an exposed skin area of approximately 3 × 5 cm^2^. Subsequently, the MCEMEA was placed on the depilated area to ensure close contact with the skin and the penetration of the stratum corneum. The MCEMEA was connected to the electrochemical workstation and a bias voltage of 0.5 V was applied to the sensing channels of the MCEMEA. For each sensing channel, amperometric signals lasting 120 s were recorded, separately (Figure S11). Blood samples were collected from the tail vein of the rats after recording and the glucose level in the blood was measured using a Roche glucometer. The measured amperometric values were converted to glucose concentrations according to the MCEMEA-current–metabolite-concentration standard curve. After the measurement, the MCEMEAs were removed and the rats were returned to their cages.

## 4. Conclusions

In this work, in response to the difficulties of microneedle sensing array fabrication, 1D and 2D microneedle electrodes were developed into MCEMEAs by the separated functionalization and assembly process, which avoided the functional coating destruction during sensor fabrication and avoided interference between adjacent electrodes during modification. The 2dMN-based electrode sheets prepared by laser cutting were developed into 2dMN-based sensing electrodes, and subsequently integrated with the 3D-printed substrate for 2dMN-based MCEA construction. The 1dMN-based MCEA was achieved by combining the laser-cut 1dMN-based electrodes with the 3D-printed resin, PI electrodes, and PDMS buffer layer for assembly, and each microneedle tip was individually modified with coating. The adjustable microneedle electrodes in the pre-position stage aid in the functionalized modification and integration, thereby reducing damage to the coating in the soldering operation. MCEMEAs showed excellent skin penetration, sensing selectivity and reproducibility in in vitro experiments, while in vivo experiments demonstrated that the MCEAs also had potential for real-time, in situ monitoring of glucose levels in living animals. The separated functionalization and assembly process could be further combined with various modification methods to enhance the multi-parameter detection capability of the sensor devices, as well as providing possibilities for the development and application of multi-channel or multi-parameter microneedle sensing systems.

## Data Availability

Data are contained within the article.

## Supplementary Materials

The following supporting information can be downloaded at: https://www.mdpi.com/article/10.3390/bios14050243/s1, Figure S1: The design of the 2dMN-based electrode. The diagram illustrates the design of the 2dMN-based electrode sheet. The width and thickness of each microneedle is 3 mm and 0.2 mm, respectively. The back of the microneedle base is equipped with a handle 15.4 mm long and 1.5 mm wide. Each 2dMN-based electrode sheet has five microneedles evenly distributed, each with a length of 0.8 mm and a maximum width of 0.4 mm. The gap between the microneedles was set at 1.2 mm. Figure S2: (a) Chronoamperometric curves depicting the current variation over time during the electrochemical gold deposition of microneedle electrodes. To enhance the surface biocompatibility and mitigate the instability of microneedle electrodes, the electrochemical deposition of gold was performed on the surface of the electrodes after fabrication and cleaning. The microneedle electrode was electrochemically deposited by CHI760E in a gold sulfite solution using a microneedle electrode as the working electrode, a commercial gold electrode as the counter electrode, and a commercial Ag/AgCl electrode as the reference electrode. Initially, a cleaning process is performed on the microneedle electrodes by applying a current of 0.6 mA for 15 s, effectively removing impurities adhering to the electrode surface. Next, further cleaning is carried out by applying a continuous current of −0.6 mA for 15 s. In the last phase, a uniform gold layer is deposited on the microneedle electrode surface by applying a current of −2 mA for 300 s, maintaining the electrode surface voltage at around −0.7 V. (b) Chronoamperometric curves illustrating the current variation over time during the electrochemical platinum deposition of gold-plated microneedle electrodes. The gold-plated microneedle electrode was electrochemically deposited by CHI760E in a platinum sulfite solution using a gold-plated microneedle electrode as the working electrode, a commercial platinum electrode as the counter electrode, and a commercial Ag/AgCl electrode as the reference electrode. Similarly, the voltage on the electrode surface is maintained at around −0.3 V at the last stage, resulting in the uniform deposition of platinum on the microneedle electrode surface. Figure S3: Current variation collected over time for microneedle electrodes modified with or without gold deposition in the PBS at a bias voltage of 0.5 V. With the presence of gold, amperometric signals collected by the microneedle electrode maintained a stable level for more than 600 s, while currents collected by the microneedle electrode without gold increased significantly at around 60 s, indicating the polarization of the electrode. The microneedle electrode modified with gold revealed higher stability under positive bias during electrochemical detection. Figure S4: The comparison of potential difference values between individual reference electrodes and the calomel electrode in the PBS. The stability of the prepared microneedle reference electrodes was tested using the open circuit voltage method. The potential difference between each prepared microneedle reference electrode and the calomel electrode was maintained at about 50 mV, suggesting that the microneedle reference electrodes modified with Ag/AgCl ink showed stable potentials during usage. Figure S5: The design schematic of the supporting structure for the 2dMN-based MCEA assembly. The figure presents the design of the 3D-printed supporting structure for the integration of the 2dMN-based MCEA. The designed support structure is 15 mm long, 15 mm wide, and 3 mm thick. The support structure has five hollow channels, each 13 mm long and 0.2 mm wide, with a 3 mm spacing. These channels facilitate the stacking of 2dMN-based electrodes to form a 2dMN-based MCEA. Figure S6: The schematic diagram of a PI thin-film circuit design for integrating a 1dMN-based MCEA. The designed PI thin-film electrode is 15 mm long, 15 mm wide, and 150 µm thick. The electrode consists of five channels with a total of nine electrode pads arranged in a 3 × 3 array. Each electrode pad within a channel has an outer diameter of 1.2 mm, an inner diameter of 0.2 mm, and a spacing of 3 mm between centers. The working electrode channel can have three 1dMN-based working electrodes connected in parallel, while both the reference electrode channel and the counter electrode channel have three electrodes connected in series, respectively. After integration with the 1dMN-based electrodes, the PI film electrodes can be connected to the PCB via an FPCB interface for stable signal transmission. Figure S7: The schematic of the flowchart of PI flexible thin-film electrodes. The electrode pattern of the PI electrode was designed using design software (AutoCAD 2018), followed by sputtering a copper thin film on the PI flexible substrate. Next, the copper film is electrochemically deposited on the substrate. Subsequently, the copper thin film on the electrode was patterned by exposure development by applying a dry film and laser imaging. The copper material was stripped from the non-target area using etching to expose the circuit pattern. Electrochemical gold deposition was performed on the patterned electrode to improve the electrode conductivity, and a cover film was pressed and fitted to the substrate. The substrate was cut and drilled to obtain the patterned PI flexible thin-film electrode. The PI flexible thin-film electrodes could also be prepared by Shenzhen Honghong Precision Circuit Co., Ltd, Shenzhen, China. Figure S8: Optical images of the PI electrode for the integration of the 1dMN-based MCEA and the 2dMN-based MCEA. (a) The optical image of a PI film electrode with nine electrode pads designed for the 1dMN-based MCEA. Each electrode pad is associated with a silver wire of about 50 µm in diameter and 7 mm in length. After separating the 1dMN-based electrodes from the fictitious ones, the silver wires were intricately wound and fixed to the 1dMN-based electrodes. (b) The optical image of the backside of the 1dMN-based MCEA. After penetrating the PI electrode, support structure, and PDMS layer, the 1dMN-based electrode is wrapped with silver wire from the PI film electrode pad. The silver wires are bonded using a silver conductive adhesive to form a stable connection. The resulting 3 × 3 1dMN-based electrode array includes three series-connected reference electrodes, three series-connected counter electrodes, and three parallel-connected working electrodes. In addition, the 1dMN-based MCEA can be reliably connected to the corresponding PCB via an FPCB interface for stable signal transmission. (c) The optical image of the 2dMN-based MCEA. Figure S9: The characterization of MCEMEA reproducibility. The as-fabricated MECMEAs were repeatedly tested in the in vitro environment and the sensitivity was statistically normalized. The detection sensitivity of each channel in both the 2dMN-based MCEA and 1dMN-based MCEA was statistically analyzed and normalized using sensitivity of day 1 as the benchmark value. Figure S10: The schematic diagram showing the characterization of MCEMEA penetration ability. In vitro experiments were performed using porcine skin to simulate human skin and we placed it on a base. The electrode tip of the MCEMEA was placed vertically downward on the surface of the porcine skin. Then, pressure was applied vertically so that the MCEMEA was pressed down uniformly, and the electrode tip was inserted vertically into the skin surface until there was complete penetration. Figure S11: The photograph showing the application of the MCEA on an anesthetized rat. The animals used included two diabetic rats. Initially, the rats were anesthetized using gas anesthesia, followed by the depilation of the rat’s back using surgical scissors and depilatory cream, resulting in an exposed skin area of approximately 3 × 5 cm^2^. Subsequently, the MCEMEA was placed on the depilated area to ensure close contact with the skin and the penetration of the stratum corneum. Figure S12: In vivo detection results of the three sensing channels in the 2dMN-based MCEA and 1dMN-based MCEA. The MCEA was inserted into the rat skin and the amperometric signals were examined over time.

## References

[1] Wang Z., Wang J., Kahkoska A.R., Buse J.B., Gu Z. (2021). Developing Insulin Delivery Devices with Glucose Responsiveness. Trends Pharmacol. Sci..

[2] Han C.S., Richley M.A. (2020). Advancing technology in diabetes management: A review of the science and technology behind current insulin pumps and CGMs for managing type 1 diabetes mellitus in pregnancy. Contemp. OB/GYN.

[3] Ma S., Li J., Pei L., Feng N., Zhang Y. (2023). Microneedle-based interstitial fluid extraction for drug analysis: Advances, challenges, and prospects. J. Pharm. Anal..

[4] Fang J., Huang S., Liu F., He G., Li X., Huang X., Chen H.J., Xie X. (2022). Semi-Implantable Bioelectronics. Nanomicro Lett..

[5] Naik A.R., Zhou Y., Dey A.A., Arellano D.L.G., Okoroanyanwu U., Secor E.B., Hersam M.C., Morse J., Rothstein J.P., Carter K.R. (2021). Printed microfluidic sweat sensing platform for cortisol and glucose detection. Lab Chip.

[6] Lin S., Cheng X., Zhu J., Wang B., Jelinek D., Zhao Y., Wu T.Y., Horrillo A., Tan J., Yeung J. (2022). Wearable microneedle-based electrochemical aptamer biosensing for precision dosing of drugs with narrow therapeutic windows. Sci. Adv..

[7] Lee I., Probst D., Klonoff D., Sode K. (2021). Continuous glucose monitoring systems—Current status and future perspectives of the flagship technologies in biosensor research. Biosens. Bioelectron..

[8] Goud K.Y., Moonla C., Mishra R.K., Yu C., Narayan R., Litvan I., Wang J. (2019). Wearable Electrochemical Microneedle Sensor for Continuous Monitoring of Levodopa: Toward Parkinson Management. ACS Sens..

[9] Xu J., Cheng C., Li X., Lu Y., Hu S., Liu G., Zhu L., Wang N., Wang L., Cheng P. (2021). Implantable platinum nanotree microelectrode with a battery-free electrochemical patch for peritoneal carcinomatosis monitoring. Biosens. Bioelectron..

[10] Lee H., Hong Y.J., Baik S., Hyeon T., Kim D.H. (2018). Enzyme-Based Glucose Sensor: From Invasive to Wearable Device. Adv. Healthc. Mater..

[11] Huang S., Yang C., Liu Z., Huang X., Chen H.-J. Wireless Implantable Phototherapy Device for Oral Inflammation Repair. Proceedings of the 2021 IEEE 16th International Conference on Nano/Micro Engineered and Molecular Systems (NEMS).

[12] Kim S., Malik J., Seo J.M., Cho Y.M., Bien F. (2022). Subcutaneously implantable electromagnetic biosensor system for continuous glucose monitoring. Sci. Rep..

[13] Tybrandt K., Khodagholy D., Dielacher B., Stauffer F., Renz A.F., Buzsaki G., Voros J. (2018). High-Density Stretchable Electrode Grids for Chronic Neural Recording. Adv. Mater..

[14] Baranwal A., Chandra P. (2018). Clinical implications and electrochemical biosensing of monoamine neurotransmitters in body fluids, in vitro, in vivo, and ex vivo models. Biosens. Bioelectron..

[15] Renard E., Riveline J.P., Hanaire H., Guerci B.J.D. (2022). Reduction of clinically important low glucose excursions with a long-term implantable continuous glucose monitoring system in adults with type 1 diabetes prone to hypoglycaemia: The France Adoption Randomized Clinical Trial. Diabetes Obes. Metab..

[16] Jia Z., Huang L., Liu H., Huang Y., Li W., Pi X., Zheng X. (2020). Design of a Real-time Self-adjusting Calibration Algorithm to Improve the Accuracy of Continuous Blood Glucose Monitoring. Appl. Biochem. Biotechnol..

[17] Battelino T., Danne T., Bergenstal R.M., Amiel S.A., Beck R., Biester T., Bosi E., Buckingham B.A., Cefalu W.T., Close K.L. (2019). Clinical Targets for Continuous Glucose Monitoring Data Interpretation: Recommendations From the International Consensus on Time in Range. Diabetes Care.

[18] Anderson S.M., Dassau E., Raghinaru D., Lum J., Brown S.A., Pinsker J.E., Church M.M., Levy C., Lam D., Kudva Y.C. (2019). The International Diabetes Closed-Loop Study: Testing Artificial Pancreas Component Interoperability. Diabetes Technol. Ther..

[19] Chmayssem A., Nadolska M., Tubbs E., Sadowska K., Vadgma P., Shitanda I., Tsujimura S., Lattach Y., Peacock M., Tingry S. (2023). Insight into continuous glucose monitoring: From medical basics to commercialized devices. Mikrochim. Acta.

[20] Aggarwal A., Pathak S., Goyal R. (2022). Clinical and economic outcomes of continuous glucose monitoring system (CGMS) in patients with diabetes mellitus: A systematic literature review. Diabetes Res. Clin. Pract..

[21] Villena Gonzales W., Mobashsher A.T., Abbosh A. (2019). The Progress of Glucose Monitoring-A Review of Invasive to Minimally and Non-Invasive Techniques, Devices and Sensors. Sensors.

[22] Kumar Das S., Nayak K.K., Krishnaswamy P.R., Kumar V., Bhat N. (2022). Review—Electrochemistry and Other Emerging Technologies for Continuous Glucose Monitoring Devices. ECS Sens. Plus.

[23] Hassan M.H., Vyas C., Grieve B., Bartolo P. (2021). Recent Advances in Enzymatic and Non-Enzymatic Electrochemical Glucose Sensing. Sensors.

[24] Kashaninejad N., Munaz A., Moghadas H., Yadav S., Umer M., Nguyen N.T. (2021). Microneedle Arrays for Sampling and Sensing Skin Interstitial Fluid. Chemosensors.

[25] Vora L.K., Moffatt K., Tekko I.A., Paredes A.J., Volpe-Zanutto F., Mishra D., Peng K., Raj Singh Thakur R., Donnelly R.F. (2021). Microneedle array systems for long-acting drug delivery. Eur. J. Pharm. Biopharm..

[26] Ren L., Liu B., Zhou W., Jiang L. (2020). A Mini Review of Microneedle Array Electrode for Bio-Signal Recording: A Review. IEEE Sens. J..

[27] Iqbal B., Ali J., Baboota S. (2018). Recent advances and development in epidermal and dermal drug deposition enhancement technology. Int. J. Dermatol..

[28] Xie L., Zeng H., Sun J., Qian W. (2020). Engineering Microneedles for Therapy and Diagnosis: A Survey. Micromachines.

[29] Alimardani V., Abolmaali S.S., Tamaddon A.M., Ashfaq M. (2021). Recent advances on microneedle arrays-mediated technology in cancer diagnosis and therapy. Drug Deliv. Transl. Res..

[30] Luo X., Yu Q., Yang L., Cui Y. (2023). Wearable, Sensing-Controlled, Ultrasound-Based Microneedle Smart System for Diabetes Management. ACS Sens..

[31] Sun X., Ji W., Zhang B., Ma L., Fu W., Qian W., Zhang X., Li J., Sheng E., Tao Y. (2022). A theranostic microneedle array patch for integrated glycemia sensing and self-regulated release of insulin. Biomater. Sci..

[32] Liu Y., Yu Q., Luo X., Yang L., Cui Y. (2021). Continuous monitoring of diabetes with an integrated microneedle biosensing device through 3D printing. Microsyst. Nanoeng..

[33] Yu J., Wang J., Zhang Y., Chen G., Mao W., Ye Y., Kahkoska A.R., Buse J.B., Langer R., Gu Z. (2020). Glucose-responsive insulin patch for the regulation of blood glucose in mice and minipigs. Nat. Biomed. Eng..

[34] Li X., Huang X., Mo J., Wang H., Huang Q., Yang C., Zhang T., Chen H.J., Hang T., Liu F. (2021). A Fully Integrated Closed-Loop System Based on Mesoporous Microneedles-Iontophoresis for Diabetes Treatment. Adv. Sci..

[35] Gao J., Huang W., Chen Z., Yi C., Jiang L. (2019). Simultaneous detection of glucose, uric acid and cholesterol using flexible microneedle electrode array-based biosensor and multi-channel portable electrochemical analyzer. Sens. Actuators B Chem..

[36] Yang J., Zheng S., Ma D., Zhang T., Huang X., Huang S., Chen H.J., Wang J., Jiang L., Xie X. (2022). Masticatory system-inspired microneedle theranostic platform for intelligent and precise diabetic management. Sci. Adv..

[37] Gowers S.A.N., Freeman D.M.E., Rawson T.M., Rogers M.L., Wilson R.C., Holmes A.H., Cass A.E., O’Hare D. (2019). Development of a Minimally Invasive Microneedle-Based Sensor for Continuous Monitoring of beta-Lactam Antibiotic Concentrations in Vivo. ACS Sens..

[38] Hoffman M.S.F., McKeage J.W., Xu J., Ruddy B.P., Nielsen P.M.F., Taberner A.J. (2023). Minimally invasive capillary blood sampling methods. Expert. Rev. Med. Devices.

[39] Vora L.K., Sabri A.H., McKenna P.E., Himawan A., Hutton A.R.J., Detamornrat U., Paredes A.J., Larrañeta E., Donnelly R.F. (2023). Microneedle-based biosensing. Nat. Rev. Bioeng..

[40] Wang J., Lu Z., Cai R., Zheng H., Yu J., Zhang Y., Gu Z. (2023). Microneedle-based transdermal detection and sensing devices. Lab. Chip.

[41] Zheng X.T., Yang Z., Sutarlie L., Thangaveloo M., Yu Y., Salleh N., Chin J.S., Xiong Z., Becker D.L., Loh X.J. (2023). Battery-free and AI-enabled multiplexed sensor patches for wound monitoring. Sci. Adv..

[42] Zhu Z., Wang J., Pei X., Chen J., Wei X., Liu Y., Xia P., Wan Q., Gu Z., He Y. (2023). Blue-ringed octopus-inspired microneedle patch for robust tissue surface adhesion and active injection drug delivery. Sci. Adv..

[43] Razzaghi M., Seyfoori A., Pagan E., Askari E., Hassani Najafabadi A., Akbari M. (2023). 3D Printed Hydrogel Microneedle Arrays for Interstitial Fluid Biomarker Extraction and Colorimetric Detection. Polymers.

[44] Teymourian H., Tehrani F., Mahato K., Wang J. (2021). Lab under the Skin: Microneedle Based Wearable Devices. Adv. Healthc. Mater..

[45] Lee K., Goudie M.J., Tebon P., Sun W., Luo Z., Lee J., Zhang S., Fetah K., Kim H.J., Xue Y. (2020). Non-transdermal microneedles for advanced drug delivery. Adv. Drug Deliv. Rev..

[46] Ingrole R.S.J., Gill H.S. (2019). Microneedle Coating Methods: A Review with a Perspective. J. Pharmacol. Exp. Ther..

[47] Wang M., Hu L., Xu C. (2017). Recent advances in the design of polymeric microneedles for transdermal drug delivery and biosensing. Lab. Chip.

[48] GhavamiNejad P., GhavamiNejad A., Zheng H., Dhingra K., Samarikhalaj M., Poudineh M. (2023). A Conductive Hydrogel Microneedle-Based Assay Integrating PEDOT:PSS and Ag-Pt Nanoparticles for Real-Time, Enzyme-Less, and Electrochemical Sensing of Glucose. Adv. Healthc. Mater..

[49] Caffarel-Salvador E., Kim S., Soares V., Tian R.Y., Stern S.R., Minahan D., Yona R., Lu X., Zakaria F.R., Collins J. (2021). A microneedle platform for buccal macromolecule delivery. Sci. Adv..

