# Supplementary material for: Fabrication of Multiple-Channel Electrochemical Microneedle Electrode Array via Separated Functionalization and Assembly Method
Source: Biosensors (Basel). 2024 May 13;14(5):243. doi: 10.3390/bios14050243 (PMC11118220; doi:10.3390/bios14050243)
Supplement: Supplementary file 1 [file biosensors-14-00243-s001.zip › biosensors-2956340-supplementary.pdf]

Supporting Information

For

Fabrication of Multiple-channel electrochemical microneedle electrode array via separated functionalization and assembly method

Xinshuo Huang[1,+], Shuang Huang[1,2,+], Shantao Zheng[1], Baoming Liang[1], Tao Zhang[2], Wan Yue[3], Fanmao Liu[4], Peng Shi[5], Xi Xie[1,*], Hui-jiuan Chen[1,*]

[1] State Key Laboratory of Optoelectronic Materials and Technologies; School of Electronics and Information Technology; Sun Yat-Sen University, Guangzhou, 510006, China.

[2] School of Biomedical Engineering, Shenzhen Campus of Sun Yat-Sen University, Shenzhen, 518107, China.

[3] School of Materials Science and Engineering, Sun Yat-Sen University, Guangzhou, 510006, China.

[4] Division of Hypertension and Vascular Diseases, NHC Key Laboratory of Assisted Circulation and Vascular Diseases (Sun Yat-sen University), The First Affiliated Hospital, Sun Yat-sen University, Guangzhou 510080, China.

[5] Department of Biomedical Engineering, City University of Hong Kong, Hong Kong SAR, China

[*] Corresponding authors, Email: xiexi27@mail.sysu.edu.cn; chenhuix5@mail.sysu.edu.cn.

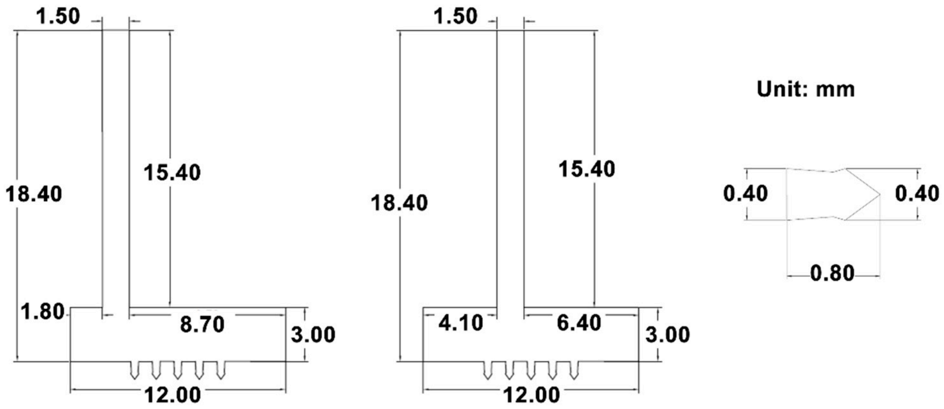

Figure S1. The design of the 2dMN-based electrode. The diagram illustrates the design of the 2dMN-based electrode sheet. The width and thickness of each microneedle is 3 mm and 0.2 mm, respectively. The back of the microneedle base is equipped with a handle 15.4 mm long and 1.5 mm wide. Each 2dMN-based electrode sheet has five microneedles evenly distributed, each with a length of 0.8 mm and a maximum width of 0.4 mm. The gap between the microneedles was set at 1.2 mm.

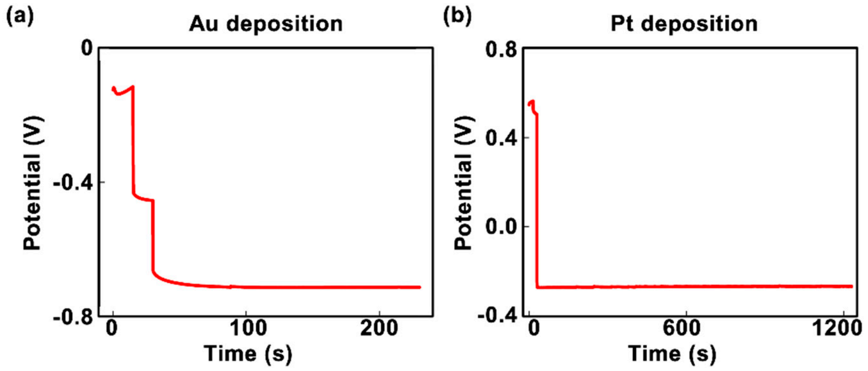

Figure S2. (a) Chronoamperometric curves depicting the current variation over time during the electrochemical gold deposition of microneedle electrodes. To enhance the surface biocompatibility and mitigate the instability of microneedle electrodes, the electrochemical deposition of gold was performed on the surface of the electrodes after fabrication and cleaning. The microneedle electrode was electrochemically deposited by CHI760E in a gold sulfite solution using a microneedle electrode as the working electrode, a commercial gold electrode as the counter electrode, and a commercial Ag/AgCl electrode as the reference electrode. Initially, a cleaning process is performed on the microneedle electrodes by applying a current of 0.6 mA for 15 seconds, effectively removing

impurities adhering to the electrode surface. Next, further cleaning is carried out by applying a continuous current of -0.6 mA for 15 seconds. In the last phase, a uniform gold layer is deposited on the microneedle electrode surface by applying a current of -2 mA for 300 seconds, maintaining the electrode surface voltage at around -0.7 V. (b) Chronoamperometric curves illustrating the current variation over time during the electrochemical platinum deposition of gold-plated microneedle electrodes. The gold-plated microneedle electrode was electrochemically deposited by CHI760E in a platinum sulfite solution using a gold-plated microneedle electrode as the working electrode, a commercial platinum electrode as the counter electrode, and a commercial Ag/AgCl electrode as the reference electrode. Similarly, the voltage on the electrode surface is maintained at around -0.3 V at the last stage, resulting in the uniform deposition of platinum on the microneedle electrode surface.

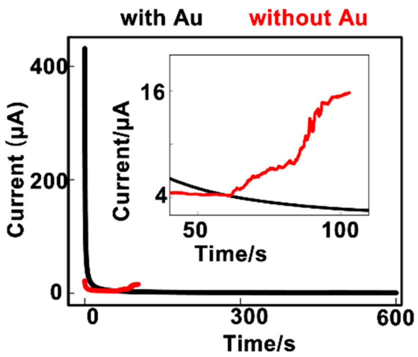

Figure S3. Current variation collected over time for microneedle electrodes modified with or without gold deposition in the PBS at a bias voltage of 0.5 V. With the presence of gold, amperometric signals collected by the microneedle electrode maintained a stable level for more than 600 s, while currents collected by the microneedle electrode without gold increased significantly at around 60 s, indicating the polarization of the electrode. The microneedle electrode modified with gold revealed higher stability under positive bias during electrochemical detection.

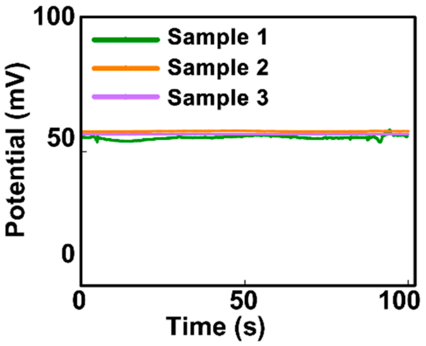

Figure S4. The comparison of potential difference values between individual reference electrodes and the calomel electrode in the PBS. The stability of the prepared microneedle reference electrodes was tested using the open circuit voltage method. The potential difference between each prepared microneedle reference electrode and the calomel electrode was maintained at about 50 mV, suggesting that the microneedle reference electrodes modified with Ag/AgCl ink showed stable potentials during usage.

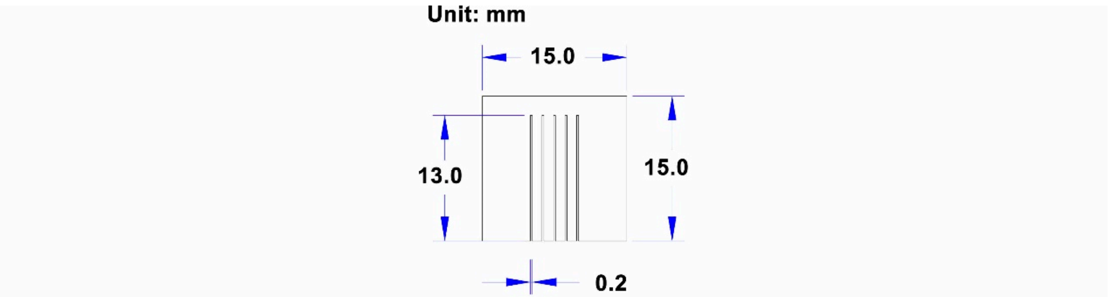

Figure S5. The design schematic of the supporting structure for the 2dMN-based MCEA assembly. The figure presents the design of the 3D-printed supporting structure for the integration of the 2dMN-based MCEA. The designed support structure is 15 mm long, 15 mm wide, and 3 mm thick. The support structure has five hollow channels, each 13 mm long and 0.2 mm wide, with a 3 mm spacing. These channels facilitate the stacking of 2dMN-based electrodes to form a 2dMN-based MCEA.

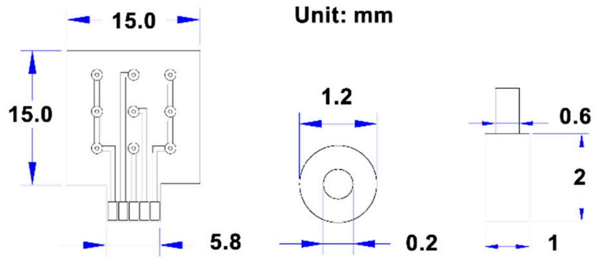

Figure S6. The schematic diagram of a PI thin-film circuit design for integrating a 1dMN-based MCEA. The designed PI thin-film electrode is 15 mm long, 15 mm wide, and 150 µm thick. The electrode consists of five channels with a total of nine electrode pads arranged in a 3x3 array. Each electrode pad within a channel has an outer diameter of 1.2 mm, an inner diameter of 0.2 mm, and a spacing of 3 mm between centers. The working electrode channel can have three 1dMN-based working electrodes connected in parallel, while both the reference electrode channel and the counter electrode channel have three electrodes connected in series, respectively. After integration with the 1dMN-based electrodes, the PI film electrodes can be connected to the PCB via an FPCB interface for stable signal transmission.

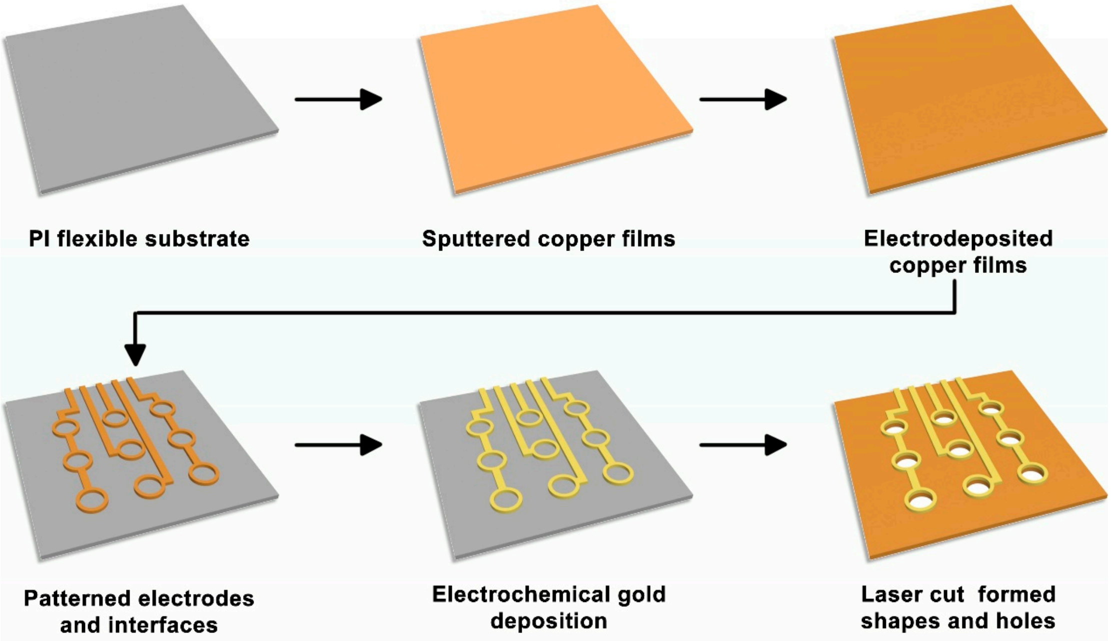

Figure S7. The schematic of the flowchart of PI flexible thin-film electrodes. The electrode pattern of the PI electrode was designed using AD software, followed by sputtering a copper thin film on the PI flexible substrate. Next, the copper film is electrochemically deposited on the substrate. Subsequently, the copper thin film on the electrode was patterned by exposure

development by applying a dry film and laser imaging. The copper material was stripped from the non-target area using etching to expose the circuit pattern. Electrochemical gold deposition was performed on the patterned electrode to improve the electrode conductivity, and a cover film was pressed and fitted to the substrate. The substrate was cut and drilled to obtain the patterned PI flexible thin-film electrode. The PI flexible thin-film electrodes could also be prepared by Shenzhen Honghong Precision Circuit co., ltd.

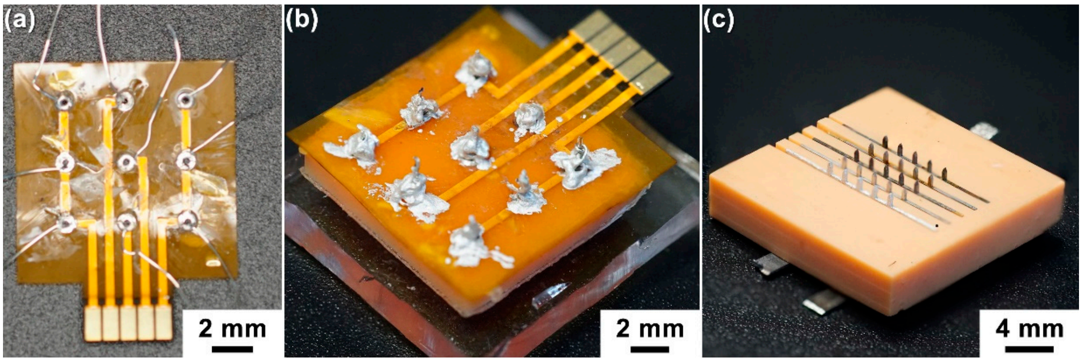

Figure S8. Optical images of the PI electrode for the integration of the 1dMN-based MCEA and the 2dMN-based MCEA. (a) The optical image of a PI film electrode with nine electrode pads designed for the 1dMN-based MCEA. Each electrode pad is associated with a silver wire of about 50 μm in diameter and 7 mm in length. After separating the 1dMN-based electrodes from the fictitious ones, the silver wires were intricately wound and fixed to the 1dMN-based electrodes. (b) The optical image of the backside of the 1dMN-based MCEA. After penetrating the PI electrode, support structure, and PDMS layer, the 1dMN-based electrode is wrapped with silver wire from the PI film electrode pad. The silver wires are bonded using a silver conductive adhesive to form a stable connection. The resulting 3x3 1dMN-based electrode array includes three series-connected reference electrodes, three series-connected counter electrodes, and three parallel-connected working electrodes. In addition, the 1dMN-based MCEA can be reliably connected to the corresponding PCB via an FPCB interface for stable signal transmission. (c) The optical image of the 2dMN-based MCEA.

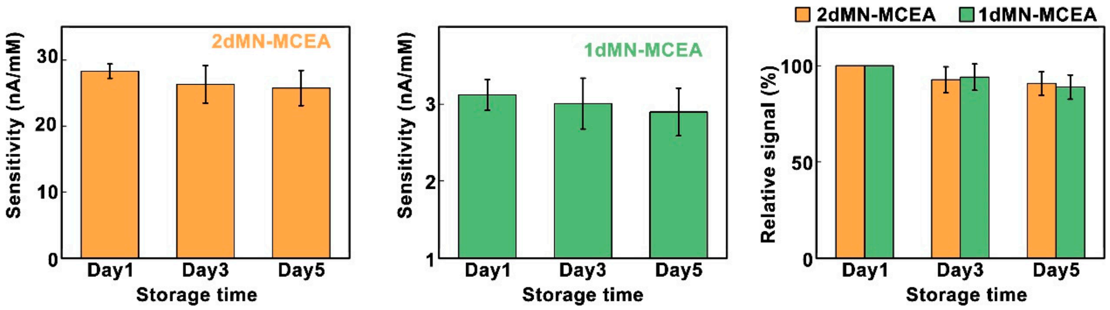

Figure S9. The characterization of MCEMEA reproducibility. The as-fabricated MECMEAs were repeatedly tested in the in vitro environment and the sensitivity was statistically normalized. The detection sensitivity of each channel in both the 2dMN-based MCEA and 1dMN-based MCEA was statistically analyzed and normalized using sensitivity of day 1 as the benchmark value.

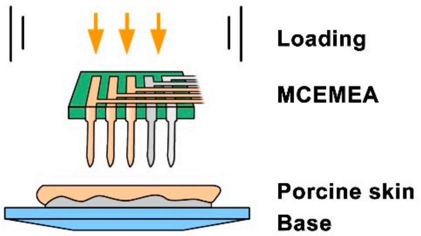

Figure S10. The schematic diagram showing the characterization of MCEMEA penetration ability. In vitro experiments were performed using porcine skin to simulate human skin and we placed it on a base. The electrode tip of the MCEMEA was placed vertically downward on the surface of the porcine skin. Then, pressure was applied vertically so that the MCEMEA was pressed down uniformly, and the electrode tip was inserted vertically into the skin surface until there was complete penetration.

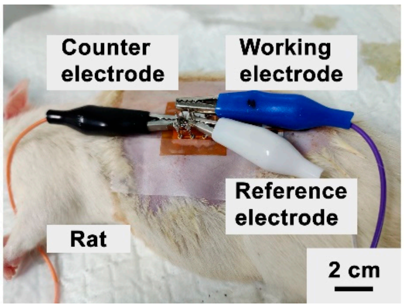

Figure S11. The photograph showing the application of the MCEA on an anesthetized rat. The animals used included two diabetic rats. Initially, the rats were anesthetized using gas anesthesia,

followed by the depilation of the rat's back using surgical scissors and depilatory cream, resulting in an exposed skin area of approximately 3×5 cm^2. Subsequently, the MCEMEA was placed on the depilated area to ensure close contact with the skin and the penetration of the stratum corneum.

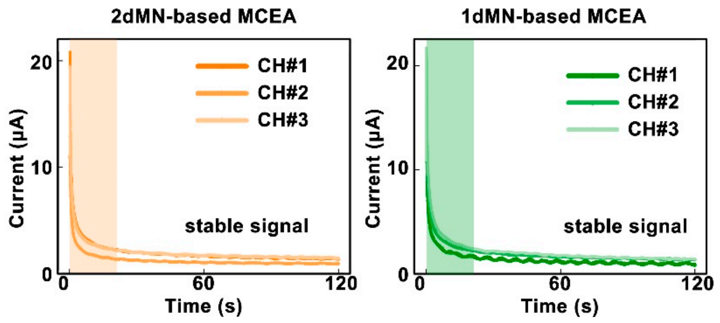

Figure S12. In vivo detection results of the three sensing channels in the 2dMN-based MCEA and 1dMN-based MCEA. The MCEA was inserted into the rat skin and the amperometric signals were examined over time.
